# Resilience and health-related quality of life in patients with pulmonary diseases receiving ambulatory oxygen therapy

**DOI:** 10.1186/s12890-021-01515-5

**Published:** 2021-05-01

**Authors:** Siiri Isokääntä, Kirsi Honkalampi, Hannu Kokki, Harri Sintonen, Merja Kokki

**Affiliations:** 1 Department of Anaesthesiology and Intensive Care, Kuopio University Hospital, Puijonlaaksontie 2, PO Box 100, 70029 Kuopio, Finland; 2 School of Medicine, Faculty of Health Sciences, University of Eastern Finland, Kuopio, Finland; 3 School of Educational Sciences and Psychology, University of Eastern Finland, Joensuu, Finland; 4 Department of Public Health, University of Helsinki, Helsinki, Finland

**Keywords:** Resilience, Psychological, Quality of life, Oxygen inhalation therapy, Lung diseases, Anxiety, Depression

## Abstract

**Background:**

Pulmonary diseases affect health-related quality of life (HRQoL), but there are few data on patients’ adaptation to a serious illness. This study assessed resilience and its associations with HRQoL, life satisfaction, anxiety and depression in patients with pulmonary diseases receiving ambulatory oxygen therapy.

**Methods:**

In this prospective cohort study, we enrolled 42 patients with pulmonary diseases receiving ambulatory oxygen therapy. The patients completed the following questionnaires at baseline and after one and three months; the Resilience Scale-25, the Life Satisfaction Scale-4, the 15D instrument of HRQoL, the Hospital Anxiety and Depression Scale (HADS) and the Quebec User Evaluation of Satisfaction with Assistive Technology (QUEST 2.0). To compare HRQoL, we recruited age- and gender-matched controls from the general population (n = 3574). The primary outcome was the proportion of patients with low resilience.

**Results:**

Half (42–48%) of the patients had low resilience, which was correlated with low HRQoL, low levels of life satisfaction and higher levels of anxiety and depression. Patients had very low HRQoL compared to controls. Dissatisfaction with life increased during the 3-months follow-up, but only a few patients had anxiety or depression. Patient satisfaction with assistive technology was high; the median QUEST 2.0 score (scale 1–5) was 4.00 at baseline, 3.92 at one month and 3.88 at three months.

**Conclusions:**

Resilience was low in half of the patients with pulmonary diseases receiving ambulatory oxygen therapy. Higher resilience was positively correlated with HRQoL and life satisfaction and negatively correlated with anxiety and depression.

*Trial registration*: ClinicalTrials.gov Protocol Record 507A023. Registered 17 September 2020—Retrospectively registered, https://clinicaltrials.gov/ct2/results?cond=&term=NCT04554225&cntry=&state=&city=&dist=.

## Background

Dyspnea is a common symptom in patients with pulmonary diseases, such as chronic obstructive pulmonary disease (COPD) [1] and interstitial lung diseases [2]. Patients with these diseases often have low health-related quality of life (HRQoL) [3, 4] and life satisfaction (LS) [5] and high levels of anxiety [6] and depression [5, 7, 8].

Oxygen therapy is used in patients with severe pulmonary diseases to improve HRQoL [9] and prolong survival [10, 11]. Ambulatory oxygen therapy is prescribed to patients who become transiently hypoxemic, e.g., during exercise or normal outdoor activities [9]. Adherence is an issue; Mesquita et al. [12] found that only 46% of patients with COPD were adherent to long-term oxygen therapy. The reasons for this low adherence include discomfort related to the nasal prongs, noise and other dissatisfaction with the assistive device [13].

Proper adaptation to a serious illness, such as advanced COPD or interstitial lung diseases, necessitates resilience. Resilience is the ability to appropriately adapt to adversities, such as health problems, traumas and other difficulties in life. This ability is based on a person’s psychological resources, and it can be learned and improved with time and effort [14]. Resilience is often evaluated in psychological examinations and research, but less attention has been paid to resilience in the medical context even though resilience capacity is closely connected to recovery abilities and adaptation [15, 16].

To the best of our knowledge, no previous studies have measured resilience in patients with pulmonary diseases receiving ambulatory oxygen therapy and how it correlates with other measures of HRQoL and LS. In this study, we evaluated the level and correlations of resilience with HRQoL, LS, anxiety and depression and examined how satisfied patients were with ambulatory oxygen therapy devices and associated services. We also compared the HRQoL of patients with pulmonary diseases receiving ambulatory oxygen therapy to an age- and gender-matched sample from the general population. The primary outcome measure was the proportion of patients with low resilience, and secondary outcomes were the correlations between resilience and HRQoL, LS, anxiety and depression in patients undergoing ambulatory oxygen therapy. Our hypothesis was that patients with pulmonary diseases requiring oxygen therapy would have low HRQoL and LS and high anxiety and depression at baseline and that patients with higher resilience would benefit more from oxygen therapy.

## Methods

### Study design and participants

The Research Ethics Committee of the Northern Savo Hospital District, Kuopio, Finland (No. 297/2017; October 10, 2017) approved the study protocol. The study had institutional approval (No. 237/2017) that was updated (No. 197/2018) after the new regulation of the European Union on the protection of natural persons with regard to the processing of personal data (2016/679). The study complies with the principles presented in the Declaration of Helsinki. Here, we report the first 3 months of follow-up data from 42 patients.

This was a prospective cohort study of patients with pulmonary diseases receiving ambulatory oxygen therapy in Finland. Patients were recruited nationwide across Finland between April 2018 and February 2020. The patients received the invitation package from the providers of the ambulatory oxygen device. It included written information about the study, a consent form, background questionnaires, baseline questionnaires and a prepaid envelope for returning the filled forms. The provider of the ambulatory oxygen device provided the invitation package only and directed any questions about the study to the researchers. The contact information for the researchers was provided, and patients were highly encouraged to contact the researchers if they had anything to discuss or wanted additional information. Patients were eligible for the study if they were prescribed ambulatory oxygen therapy because of a pulmonary disease and were aged 18 years or older. We did not enroll patients undergoing oxygen therapy for indications other than pulmonary diseases.

Patients who agreed to participate in the study completed the questionnaires themselves at home at baseline and after one and three months. The baseline questionnaires comprised questions about background information, and at all three time points, the patients completed questionnaires assessing resilience, HRQoL, LS, anxiety, depression and satisfaction with assistive technology. The second and third sets of questionnaires were mailed to the patients to be returned in a prepaid envelope. Those who had not responded within two weeks were contacted and/or interviewed by phone. After the patients gave written consent, medical records concerning oxygen therapy were obtained from the hospitals that had prescribed oxygen therapy.

The participants completed five questionnaires at each time point. The *Resilience scale-25 *(RS-25, [15, 17]) assesses resilience with 25 statements that measure subjective and interpersonal protective resources that help in adaptation to adversities. The RS-25 is a self-report measure with each item scored on a 7-point Likert scale from 1 (strongly disagree) to 7 (strongly agree). Total scores range between 25 and 175: scores ranging from 25–100 indicate very low resilience, scores from 101–115 indicate low resilience, scores from 116–130 indicate moderately low resilience, scores from 131–145 indicate moderate resilience, scores from 146-160 indicate moderately high resilience, and scores from 161–175 indicate high resilience. We dichotomized the resilience scores into two groups: scores from 25–130 indicate low resilience, and scores from 131–175 indicate moderate to high resilience [17]. Internal consistency with the current sample was excellent at each time point (Cronbach’s alpha = 0.940).

Health-related quality of life was measured by the *15D instrument*. 15D is a generic, 15-dimensional, standardized, self-administered instrument that can be used both as a profile and a single index score measure. The health state descriptive system (questionnaire) is composed of the following dimensions: mobility, vision, hearing, breathing, sleeping, eating, speech (communication), excretion, usual activities, mental function, discomfort and symptoms, depression, distress, vitality, and sexual activity, with five levels for each dimension. The single index score (15D score), representing the overall HRQoL on a scale from 0 to 1 (1 = full health, 0 = being dead), and the dimension level values, reflecting the goodness of the levels relative to no problems on the dimension (= 1) and to being dead (= 0), are calculated from the questionnaire by using a set of population-based preference or utility weights. Mean dimension level values are used to create 15D profiles for groups [18]. The minimum clinically important difference in the 15D score is ± 0.015 [19]. In the current sample, the internal consistency was good at each time point (Cronbach’s alpha = 0.859).

The HRQoL of patients was compared with that of a sample from the general Finnish population. The 15D data for the general population came from the National Health 2011 Health Examination Survey representing the Finnish population aged 18 years and older [20]. For this analysis, we stratified the individuals by age and gender. The individuals selected (n = 3574) were in the same age range (48–89 years) as the patients, and was matched with the gender distribution with the present study population.

*The Life Satisfaction Scale-4 *(LS-4, [21]) measures LS based on four items that measure interest and happiness in life, ease of living, and loneliness. The LS-4 is a 4-item self-report measure, and the items are scored on a 5-point Likert scale of decreasing LS. The total score varies from 4 to 20; scores ranging from 4 to 6 indicate that the respondent is satisfied with life, scores from 7 to 11 indicate that the respondent is slightly dissatisfied, and scores from 12 to 20 indicate that the respondent is dissatisfied. We dichotomized the data into two groups: scores from 4 to 11 indicate that the respondent is satisfied with life, and scores from 12 to 20 indicate that the respondent is dissatisfied with life. In the current sample, the internal consistency was good at each time point (Cronbach’s alpha = 0.841).

*The Hospital Anxiety and Depression Scale* (HADS, [22]) assesses anxiety and depressive symptoms. The HADS is a 14-item self-report measure with a 4-point scale of increasing anxiety or depression. Uneven numbered questions assess anxiety, and even-numbered questions assess depression. For both dimensions, scores of 0–7 indicate non-cases, scores of 8–10 indicate mild anxiety, scores of 11–14 indicate moderate anxiety, and scores of 15–21 indicate severe anxiety or depression. We dichotomized the data into two groups: scores of 0–10 indicate no or mild symptoms, and scores of 11–21 moderate to severe anxiety or depression. Internal consistency with the current sample was excellent at each time point (Cronbach’s alpha = 0.912).

The *Quebec User Evaluation of Satisfaction with Assistive Technology 2.0 *(QUEST 2.0, [23]) measures satisfaction with assistive technology. QUEST 2.0 is a 12-item self-report measure with a 5-point Likert scale of increasing satisfaction with assistive technology. The QUEST 2.0 data are often dichotomized; scores of 1–3 indicate dissatisfaction, and scores of 4–5 indicate satisfaction. We dichotomized the data into two groups based on average scores: ≤ 3 indicates dissatisfaction, and > 3 indicates satisfaction. Questions 1–8 assess satisfaction with assistive technology devices, and questions 9-12 assess satisfaction with assistive technology services. For QUEST 2.0, we calculated the average of both sections and the total score. Furthermore, QUEST 2.0 has a list of 12 components of satisfaction, from which the patient selects the three most important factors for them. Internal consistency with the current sample was excellent at each time point (Cronbach’s alpha = 0.926).

### Outcome measures

The primary outcome measure was the proportion of patients with low resilience (RS-25 ≤ 130), and the secondary outcomes were the correlations between resilience and HRQoL, LS, anxiety and depression in patients prescribed ambulatory oxygen therapy.

### Statistics

The data were entered and analyzed with SPSS software (IBM SPSS Statistics 25, International Business Machines Corporation, Armonk, NY, USA). The normality of the distributions of continuous variables was tested with the Kolmogorov–Smirnov test, the Shapiro–Wilk test and visual inspection of the histograms. We analyzed the continuous variables with the Wilcoxon signed-rank and the Friedman test, and binominal and categorical variables with the Cochran Q and the Chi-square test. Correlations between variables were evaluated with Spearman's rank correlation test. Cronbach’s alpha was used to measure the internal consistency for each questionnaire at every time point. The data are presented as number of cases, mean (SD), median (minimum–maximum) and 95% confident intervals (CI) as appropriate. *P* values ≤ 0.05 were considered statistically significant.

## Results

**Fig. 1:**
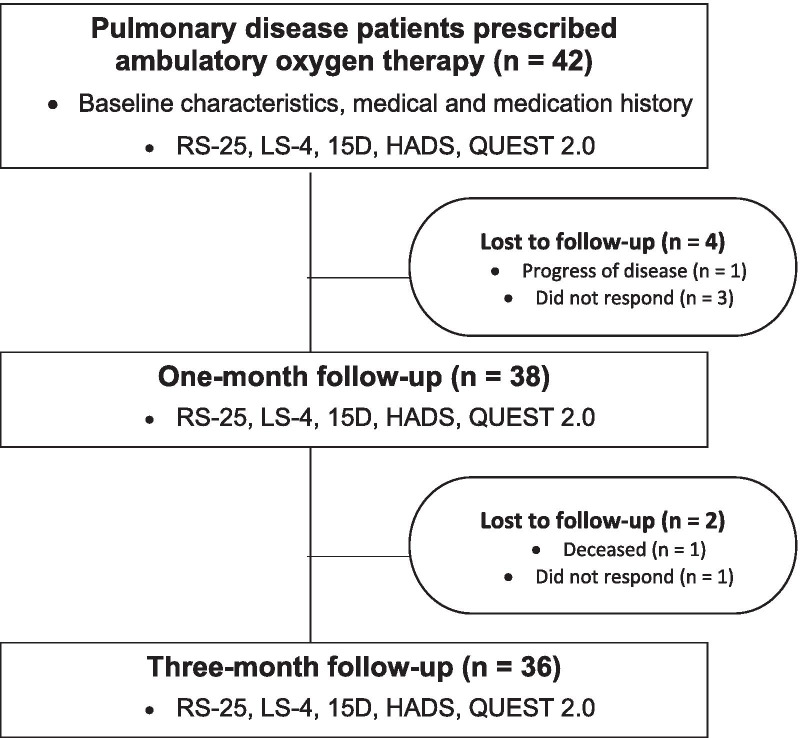
Flow chart of participants. RS-25 = Resilience Scale-25; LS-4 = Life Satisfaction Scale-4; 15-D = 15-D Instrument of Health-related Quality of Life; HADS = Hospital Anxiety and Depression Scale; QUEST 2.0 = Quebec User Evaluation of Satisfaction with Assistive Technology

Our aim was to collect a study sample of 100 patients with ambulatory oxygen therapy within 12 months, but due to a slow recruitment of patients and the outbreak of the Covid-19 pandemic, we decided to stop enrolment in March 2020. Thus, we have data on 42 patients at baseline, 38 patients at one month (a response rate of 90%), and 36 patients at three months (a response rate of 86%). Figure 1 shows the flow chart.

The patients’ baseline characteristics are shown in Table 1. Most patients were men (n = 32), and 28 (67%) had COPD. Four men had two diagnoses of pulmonary diseases: two of them had both COPD and pulmonary fibrosis; one had pulmonary fibrosis, asbestos lung disease and pleural plaques; and one had both COPD and asbestos. One woman had both pulmonary fibrosis and asthma. All patients had concomitant diseases, with cardiovascular (n = 33) and endocrine and metabolic diseases (n = 26) being the most common. Twelve patients had oxygen therapy before initiation of ambulatory oxygen therapy and 30 patients had no previous oxygen therapy.

**Table 1:** Baseline participant characteristics. Data are the number of cases or median (minimum–maximum)

Variable	All participants (N = 42)
Sex, male/female (n)	32/10
Age (years) (median (min–max))	72 (48–89)
BMI (kg/m^2^) (median (min–max))	27 (14–57)
Height (cm) (median (min–max))	170 (149–188)
Weight (kg) (median (min–max))	74 (47–200)
Primary pulmonary diagnosis	
COPD (n)	28
Fibrosis (n)	12
Asbestos lung disease and pleural plaques (n)	1
Alveolar hypoventilation (n)	1
Oxygen therapy before ambulatory oxygen (yes/no)	12/30
Assistive technology (yes/no)	20/22
Cane/crutches/walker (n)	18
Wheelchair (n)	5
Comorbidities, ICD-10 group (median (min–max))	5 (1–10)
IX: Cardiovascular (n)	33
IV: Endocrine and metabolic (n)	26
X: Other respiratory (n)	19
XIII: Musculoskeletal (n)	13
Other (II/VI/VII/VIII/XI/XII/XIV) (n)	7/10/6/1/5/6/5
Education	
Less than high school graduate (n)	17
High school graduate (n)	18
More than high school (n)	7
Employed/Retired	3/39
Smoking	
Current (n)	1
Former (n)	34
Never (n)	7
Smoking pack-years (median (min–max))	30 (2–150)
Age of starting smoking (yrs.)	20 (9–34)
Mortality during the three-month follow-up (n)	2

BMI, body mass index; ICD-10, International Statistical Classification of Diseases and Related Health Problems 10th Revision

Median (minimum–maximum) scores and the dichotomized data of study variables are presented in Table 2.

**Table 2:** Survey scores at baseline and after one and three months

Variable	Resilience (RS-25) Scale 25–175	Life satisfaction (LS-4) Scale 4–20	Health-related quality of life (15D) Scale 0.0–1.0	Anxiety (HADS) Scale 0–21	Depression (HADS) Scale 0–21	Satisfaction with assistive technology (QUEST 2.0) Scale 1–5
Baseline (n = 42)						
Median (min–max)	133 (75–169)	9 (5–20)	0.748 (0.414–0.946)	5 (0–16)	5 (0–16)	4.00 (1.00–5.00)
Dichotomized^a^ yes/no (n)	21/19	31/11	NA	5/37	7/35	35/5
One month (n = 38)						
Median (min–max)	132 (42–168)	9 (5–18)	0.725 (0.473–0.880)	5 (0–15)	5 (0–15)	3.92 (2.20–5.00)
Dichotomized^a^ yes/no (n)	20/17	25/13	NA	5/31	5/31	32/5
Three months (n = 36)						
Median (min–max)	134 (87–164)	10 (5–20)	0.740 (0.461–0.913)	6 (0–17)	6 (0–15)	3.88 (2.70–5.00)
Dichotomized^a^ yes/no (n)	21/15	19/17	NA	3/33	3/33	32/4

RS-25, Resilience Scale-25; LS-4, Life Satisfaction Scale-4; 15D, 15-Dimensional Health-related Quality of Life (HRQoL) instrument; HADS, Hospital Anxiety and Depression Scale; QUEST 2.0, Quebec User Evaluation of Satisfaction with Assistive Technology (Version 2.0)

^a^Dichotomized: RS-25: > 130/ ≤ 130; LS-4: 4–11/12–20; Anxiety: 0–10/11–21; Depression: 0–10/11–21; Satisfaction with assistive technology: > 3/1–3

*Low resilience* was common in patients with pulmonary diseases prescribed ambulatory oxygen therapy, and no change in resilience was observed during the three-month follow-up; at baseline, one month, and three months, 19 out of 40 (prevalence 48%; 95% CI [32, 63%], 17/37 (46%), [30, 62%], and 15/36 patients (42%, [26, 58%], p = 0.872) had an RS-25 score ≤ 130, respectively.

**Fig. 2:**
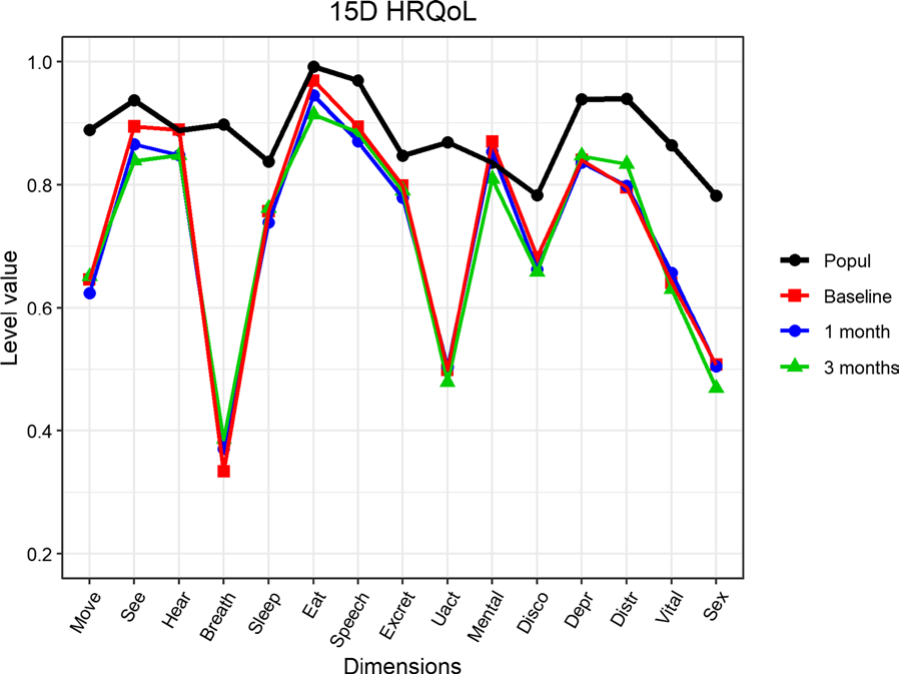
Health-related quality of life of the population and pulmonary disease patients at baseline, and after one and three months. Move, moving; See, Seeing; Hear, Hearing; Breath, breathing; Sleep, sleeping; Eat, eating; Speech, speech; Excret, excretion; Uact, usual activities; Mental, mental function; Disco, discomfort and symptoms; Depr, depression; Distr, distress; Vital, vitality and Sex, sexual activity, Popul, age- and gender-matched control population

*The health-related quality of life* of patients with pulmonary diseases was much worse (15D score difference < 0.035) at all time points than that of the age- and gender-matched sample from the general population [20] (p < 0.001, Fig. 2). The control group had a mean 15D score of 0.885 [20]; the 15D score in our sample at baseline, one month, and three months was 0.728 (mean difference compared to the population: − 0.157, [− 0.120, − 0.194]), 0.714 (− 0.171, [− 0.135, − 0.207]) and 0.707 (− 0.178, [− 0.136, − 0.220]), respectively. HRQoL had decreased at three months compared to the baseline, with a mean difference of − 0.024 [0.002, − 0.051], (p = 0.077) between the baseline and three months.

*Life satisfaction* decreased during the three-month follow-up. The proportion of patients satisfied with life (LS-4 score from 4–11) decreased from 31 out of 42 patients at baseline to 25 out of 38 at one month and to 19 out of 36 patients at three months (p = 0.151).

*Anxiety and depression* were rather uncommon considering that the HRQoL and LS were low. At baseline, five patients had HADS anxiety scores ≥ 11, indicating moderate to high anxiety; at one month and three months, five patients and three patients had HADs scores ≥ 11, respectively (p = 0.793). For depression at baseline, seven patients scored ≥ 11 points, indicating moderate or severe depression; at one month and three months, five patients and four patients had high depression scores, respectively (p = 0.770).

Resilience had moderate positive correlation with HRQoL and QUEST 2.0 scores and negative correlation with anxiety, depression and LS scores, indicating that more resilient patients have higher LS, while those with less resilience have higher levels of anxiety and depression (Table 3). In a post hoc analysis, resilience did not correlate with age (0.076, p = 0.641) or sex (− 0.024, p = 0.885).

**Table 3:** Spearman correlation coefficient between resilience and other measures

Variable	Health-related quality of life (15D)	Life satisfaction (LS-4)	Depression (HADS)	Anxiety (HADS)	Satisfaction with assistive technology (QUEST 2.0)
Baseline (n = 42)					
Spearman correlation coefficient	0.548	− 0.655	− 0.698	− 0.465	0.100
*p* value	< 0.001	< 0.001	< 0.001	0.002	0.546
One month (n = 38)					
Spearman correlation coefficient	0.437	− 0.410	− 0.477	− 0.468	0.276
*p* value	0.007	0.012	0.003	0.004	0.104
Three months (n = 36)					
Spearman correlation coefficient	0.550	− 0.661	− 0.801	− 0.678	0.110
*p* value	0.001	< 0.001	< 0.001	< 0.001	0.524

15D, 15-Dimensional Health-related Quality of Life (HRQoL) instrument; LS-4, Life Satisfaction Scale-4; HADS, Hospital Anxiety and Depression Scale; QUEST 2.0, Quebec User Evaluation of Satisfaction with Assistive Technology (Version 2.0)

*Total satisfaction with assistive technology* was high; at baseline the median QUEST 2.0 score was 4.00, and at three months it was 3.88 (p = 0.687) (Table 2). The dichotomized scores were similar to medians: at baseline, five patients were dissatisfied with the assistive technology; at one month, seven patients were dissatisfied; and at three months, four patients were dissatisfied (p = 0.598). Satisfaction with the services was higher than satisfaction with the device: at baseline, the median QUEST 2.0 score for services was 4.50 (minimum–maximum; 1.00–5.00) vs. 3.94 for the device (1.00–5.00) (p < 0.001). At one month the median score was 4.25 for services (1.80–5.00) vs. 3.75 for the device (2.40–5.00) (p = 0.046); and at three months the score was 4.00 for the services (2.30–5.00) (p = 0.121 for repeated measurements) vs. 3.75 for the device (2.40–5.00) (compared to services, p = 0.040; for repeated measurements, p = 0.836). The four most commonly selected satisfaction items were that the device was easy to use, effective, safe and its ease of adjustment.

## Discussion

The novelty of the present study was that we evaluated resilience for the first time among patients with pulmonary diseases receiving long-term oxygen therapy and examined how resilience may affect HRQoL. In our study, half of the patients had low resilience. This is important, as low resilience was associated with low HRQoL, lower levels of LS, and high levels of anxiety and depression. Our data indicate that patients starting to use ambulatory oxygen therapy were generally satisfied with the assistive technology, and satisfaction with the services was even higher. Taken together, these data indicate that among patients prescribed long-term oxygen therapy, those with low resilience should be identified and given psychological support to enhance their resilience [14].

Resilience is based on a person’s psychological resources. However, resilience is a skill that can be learned and improved with time and effort. Psychological resources are required to promote successful ageing. Successful ageing can be described as maintaining and increasing good physical and mental functioning, but in the elderly, it also comprises LS, social functioning and participation. Resilience, optimism, and coping skills are important contributors to successful ageing despite physical disabilities. Subjective health is not only the absence of diseases [24]. Resilience can buffer the negative effects of an illness [25]. Shen et al. [26] found that higher resilience is associated with reduced mortality among the elderly, even after controlling for sociodemographic characteristics and initial health status. In summary, resilience is influenced by health status, but as it is modifiable, patients with low resilience should be identified and given appropriate psychological support to enhance their adaption to the adversity caused by pulmonary diseases.

In the present study, the mean resilience score was between 130 and 132, which is substantially lower than that found in a Nordic population study of people aged between 60 and 90 years old (mean scores between 143–144) [27]. Resilience scores were steady over the three-month follow-up, which was excepted, since improving resilience requires time and persistence [14]. Resilience has been shown to be correlated with age in previous studies [27] but this correlation was not observed in our sample, presumably because of the small sample size, high median age and narrow age range. In a study by Lundman et al. [27], the covered age range was between 19 and 103, and for every one year increase of age, the RS-25 score increased by 0.134 units. Future research should focus on discerning the level of resilience in different populations with pulmonary diseases and to evaluate how they can effectively improve their resilience.

Consistent with published data on patients with COPD [3], the patients prescribed ambulatory oxygen therapy had a much lower HRQoL than the age- and gender-matched sample, and based on 15D scores, HRQoL decreased during the three-month follow-up. This is assumed to indicate disease progression and its negative effects on HRQoL. Life satisfaction was also low, which is similar to previous findings [5, 28]. Similar to HRQoL, LS also decreased during the three-month follow-up. Pulmonary diseases cause dyspnea, which may further decrease physical activity, deteriorate other daily activities and thus considerably worsen HRQoL and LS. A pulmonary disease requiring ambulatory oxygen therapy is at an advanced stage, and patients with these diseases often have several comorbidities. Thus, a decrease in HRQoL is expected. Satisfaction with the oxygen therapy device can affect perceived HRQoL [29]. Therefore, appropriate information about the oxygen therapy device could support patients’ HRQoL. In our study, satisfaction with assistive technology services was high, which indicates that the services were sufficient. Further long-term studies should explore whether reinforcing resilience could help to preserve or increase HRQoL in these patients.

The prevalence of severe anxiety (12%) and severe depression (17%) at baseline in our sample was two- to three-fold higher than that among the general population in Finland. The annual incidence of anxiety disorders in the general population in Finland is 4% and that of depression is 6.5% [30]. In the present study, two patients with anxiety at baseline did not report anxiety during the follow-up, while one patient developed anxiety. Two depressive patients at baseline did not have depressive symptoms after the initiation of ambulatory oxygen. Distress levels and depression measured with 15D were substantially higher in our sample compared to the general population, which supports the claim that patients with pulmonary diseases have a higher prevalence of mental health disorders. The size of the study sample was regrettably small, but as a pilot data it is assumed appropriate for the purpose of our analyses. Furthermore, our data are consistent with previous publications even with the inclusion of both COPD and interstitial fibrosis patients, indicating that anxiety and depression are common in this patient group [5, 28, 29, 31] and are inversely related to HRQoL [7, 31]. Furthermore, in a few patients with depressive symptoms, ambulatory oxygen therapy may lift their mood [29]. As a result, patients should be systematically screened for anxiety and depression when prescribing ambulatory oxygen therapy, and patients with mental health issues should be provided appropriate psychological support, treatment and follow-up.

There is no consensus regarding when patients should be prescribed ambulatory oxygen therapy. The British Thoracic Society [9] and Global Initiative for Chronic Obstructive Lung Disease (GOLD 2017) [32] recommend that patients with COPD having a resting stable peripheral capillary oxygen saturation (SpO_2_) of ≤ 88–92% should be referred for a blood gas assessment to assess eligibility for long-term oxygen therapy. Furthermore, assessment should also be made with SpO_2_ of ≤ 94% if patients with COPD have peripheral edema, polycythemia (hematocrit ≥ 55%) or pulmonary hypertension. Many patients with pulmonary diseases, including COPD and interstitial fibrosis, started their long-term oxygen therapy with ambulatory oxygen. British Thoracic Society guidelines recommend that ambulatory oxygen therapy should be offered to patients already on long-term oxygen therapy who are mobile outdoors [9]. The 6-min walking distance (6MWD) test can discern whether patients with COPD with a partial pressure of oxygen (PO_2_) between 60 and 70 mmHg desaturate (SpO_2_ ≤ 90%) early or late during their daily living activities. In the García-Talavera et al. study, patients who desaturated after 3.5 min of the 6MWD test did not desaturate in the 24-h pulse oximetry, but those who desaturated during the first minute had a 74% probability of desaturating in their daily activities [33]. Even though the criteria are relatively clear for starting long-term oxygen therapy, ambulatory oxygen has been prescribed without proper patient assessment, counselling, support and follow-up. Thus, patients may use it differently than aimed therapy, resulting in disappointments about the effectiveness of ambulatory oxygen therapy [34].

## Limitations

Our study has some limitations. This framework solely pertains to patients with pulmonary diseases prescribed ambulatory oxygen. The major limitations of the study are a small sample size and different history of home oxygen therapy. At baseline 12 patients were on oxygen therapy and majority, 30 patients were newly starters with no previous oxygen therapy, and this might have introduced some bias in our results. Hence, this study should be considered as a pilot study of resilience in pulmonary disease patients receiving ambulatory oxygen therapy. Further long-term studies should be conducted measuring resilience and HRQoL in different pulmonary diseases and different history of long-term oxygen therapy use. Due to 30 patients being new starters with no previous oxygen therapy, our sample might be less morbid than the average patients with pulmonary diseases receiving long-term oxygen therapy.

Second, all patients had comorbidities, and cardiovascular diseases were the most common. In our data, it is impossible to distinguish the impact of pulmonary diseases and other health issues on participants’ HRQoL and other parameters measured in the present study. Third, missing information existed in excess about patients’ physical performance, level of hypoxemia and hours of oxygen therapy used per day; therefore, we could not include them in our analyses. One of the main limitations was a small sample size, but difficulties in enrolling patients have also been common in other studies. Our aim was to enroll 100 patients, but due to low response rates that are common in pulmonary disease patients [35] and the outbreak of COVID-19, we decided to stop enrolling in March 2020. Finally, we used self-administered questionnaires and the interpretation of survey questions and rating scales can vary between individuals. Nonetheless, we used commonly used, validated questionnaires with closed-end questions that are easier to recall for the respondents than open-end questions. Moreover, self-administered questionnaires are less likely to interviewer bias in this kind of studies where sensitive issues are evaluated. Our study consisted of three repeated measurements, and adherence was high, thus decreasing the likelihood of compliance bias and supporting that our data is soundly based.

## Conclusions

In our data resilience was low in half of the patients with pulmonary diseases undergoing ambulatory oxygen therapy and HRQoL of these patients was much worse than that of the general population. This is important knowledge, as higher resilience was positively correlated with HRQoL and LS, and negatively correlated with anxiety and depression. Patients with low resilience should be identified and given psychological support to enhance their resilience, thus helping them to better adapt to adversity.

## Data Availability

Metadata is available from the corresponding author on reasonable request.

## Abbreviations

6MWD: 6-Min walking distance
15D: 15D quality of life questionnaire
COPD: Chronic obstructive pulmonary disease
GOLD 2017: Global Initiative for Chronic Obstructive Lung Disease
HADS: The Hospital Anxiety and Depression Scale
HRQoL: Health-related quality of life
LS: Life satisfaction
LS-4: The Life Satisfaction Scale-4
PO_2_: Partial pressure of oxygen
QUEST 2.0: The Quebec User Evaluation of Satisfaction with Assistive Technology 2.0
RS-25: Resilience scale-25
SpO_2_: Peripheral capillary oxygen saturation

## Code availability

The data was analyzed with the SPSS software (IBM SPSS Statistics 25, International Business Machines Corporation, Armonk, NY, USA).
